# Editorial: Assessing the environmental impact of ruminants: mitigation strategies and climate change implications

**DOI:** 10.3389/fmicb.2026.1883961

**Published:** 2026-06-04

**Authors:** Anusorn Cherdthong, Suban Foiklang, Eric Altermann

**Affiliations:** 1 Tropical Feed Resource Research and Development Center (TROFREC), Department of Animal Science, Faculty of Agriculture, Khon Kaen University, Khon Kaen, Thailand; 2 Faculty of Animal Science and Technology, Maejo University, Chiang Mai, Thailand; 3 School of Veterinary Science, Massey University, Palmerston North, New Zealand; 4 Blue Barn Life Sciences Ltd, Feilding, New Zealand

**Keywords:** by-product, environmental impact, feed addictive, greenhouse gases, methane emissions, microbiome, mitigation strategies for ransomware attacks, ruminants

Ruminants support food security by converting human-inedible biomass and agricultural by-products into nutrient-dense foods, with growing relevance to circular bioeconomy approaches (Kebreab et al., in press). At the same time, cattle, buffalo, sheep, and goats contribute substantially to agricultural greenhouse gas emissions, mainly through enteric methane production and manure-derived emissions (Wanapat et al., 2024). Methane is produced mainly through enteric fermentation in the rumen and hindgut. Fermentative bacteria, protozoa, fungi, and methanogenic archaea interact to regulate hydrogen production and disposal, volatile fatty acid production, nutrient use, and energy loss. Therefore, reducing methane emissions from ruminants is not only an environmental priority but also a nutritional and biological challenge. Ideally, methane mitigation strategies are confirmed in well-designed animal studies and account for diet, methane measurement, animal performance, health, and product quality (Hristov et al., 2025; Dijkstra et al., 2025). This Research Topic brings together 13 articles on microbial mechanisms, dietary interventions, feed additives, by-product utilization, host and management factors, and climate vulnerability in ruminant systems.

## Dietary strategies and rumen microbial modulation

Dietary manipulation remains one of the most practical routes for changing rumen fermentation because it directly alters substrate supply for bacteria, protozoa, fungi, and methanogenic archaea. The studies in this area show that forage type, feed processing, and roughage inclusion can alter methanogen composition and rumen microbial balance, although these changes do not always translate directly into confirmed methane mitigation. Dwan et al. investigated the effects of sward type and dairy cow breed on the rumen microbiome. Their findings showed that increasing sward diversity had limited effects on the core rumen microbiome, although it altered some methanogenic taxa. Crossbreeding Holstein-Friesian cows with Jersey also influenced microbial configuration, suggesting that both pasture system and host background may shape methanogen composition.

Rugwete et al. examined sorghum-based diets in Mashona goats and showed that methanogenic archaea responded differently to sorghum processing methods. Although microbial diversity was not significantly altered, malted sorghum produced a more diverse archaeal profile, with evidence of both hydrogenotrophic and methylotrophic methanogenic niches. The authors rightly emphasized the need for direct *in vivo* methane measurements to confirm whether these compositional changes translate into mitigation.

Zhang et al. studied cotton straw inclusion in total mixed pelleted feed for breeding ewes. Their work showed that cotton straw modified rumen bacterial and fungal communities, with 30% inclusion suggested as the optimal level. Higher inclusion, especially above 40%, increased Proteobacteria, indicating potential disturbance of rumen microbial balance. This finding is important in a wider application, because it highlights that the use of low-cost roughage resources must be balanced with rumen stability.

## Feed by-products and circular feeding systems

Circular livestock production depends on using feeds that reduce competition with human food while maintaining animal performance (Wanapat et al., 2024). The by-product studies in this Research Topic show that brewery waste, mushroom residue, and rose pomace can contribute to resource-efficient feeding systems. Their value, however, depends on appropriate inclusion levels and clear evidence of benefits for fermentation, digestibility, performance, or product quality. Prajapati et al. evaluated oat brewery waste as a partial concentrate replacement in growing sheep. Replacing concentrate with 20% oat brewery waste reduced daily enteric methane emissions by 14%−15.5% without impairing intake, digestibility, or growth performance. The reduction appeared to be linked to changes in protozoal populations and methylotrophic methanogen activity rather than broad restructuring of the archaeal community.

Long et al. investigated *Flammulina velutipes* mushroom residue in fermented total mixed rations for Guizhou black goats. Inclusion of 30% mushroom residue improved apparent digestibility, average daily gain, feed conversion ratio, immunity-related indicators, and economic return. Although rumen fermentation parameters and phylum-level microbial composition were not markedly changed, genus-level shifts were observed and may be related to improved digestibility and immune responses. This study supports the value of feed by-products in circular livestock production.

He et al. evaluated rose pomace supplementation in Hu sheep. Dietary inclusion of 2%−4% rose pomace improved rumen fermentation and increased beneficial fatty acids in the longissimus dorsi muscle. This work suggests that plant-derived by-products can serve not only as alternative feed resources but also as functional additives for improving rumen fermentation and meat quality.

## Bioactive compounds and targeted methane inhibition

A second route to methane mitigation is the use of bioactive compounds that suppress methanogens, redirect hydrogen, or inhibit key biochemical steps in methane formation. The papers in this group show that tannins, seaweed, and monolaurin-based additives can reduce methane-related outputs through different microbial pathways. At the same time, they highlight an important caution: anti-methanogenic effects must be evaluated alongside digestibility, intake, fermentation efficiency, and animal performance. Cremonesi et al. examined quebracho condensed tannin extract in dairy goats. Increasing tannin inclusion reduced *Methanobrevibacter* and altered bacterial, fungal, and protozoal communities. These microbial shifts were associated with lower methane production, modified volatile fatty acid profiles, and altered nutrient digestibility. However, the study also showed that high tannin levels may reduce feed conversion efficiency, reminding us that methane mitigation must not come at the cost of poor substrate utilization.

Li et al. investigated *Asparagopsis taxiformis* using *in vitro* rumen fermentation, metagenomics, and metabolomics. Freeze-dried *Asparagopsis*, particularly at 5% inclusion, strongly reduced methane production while maintaining a better balance with rumen fermentation than higher inclusion. The mechanism involved suppression of Methanobrevibacter, changes in propionate-producing bacteria, reduced hydrogen availability, and inhibition of methyl-coenzyme M reductase-related pathways. This paper provides mechanistic evidence for seaweed-based methane mitigation, although long-term animal trials remain essential.

Park et al. tested MGOsyn, a monolaurin-based additive containing garlic and oregano, using rumen fluid from Hanwoo steers. MGOsyn reduced methane production by shifting fermentation toward propionate and increasing bacteria involved in hydrogen-sink pathways, including Succinivibrio, Succiniclasticum, and Prevotella-related groups. These findings are relevant because effective methane mitigation often depends on redirecting hydrogen toward useful fermentation products rather than simply suppressing methanogens.

Shao et al. reviewed methyl-coenzyme M reductase as a target for inhibition of methanogenesis. This review places methane mitigation in a biochemical context by focusing on the final enzymatic step of methane formation. The authors discussed inhibitors such as 3-nitrooxypropanol and bromoethanesulfonate, as well as emerging approaches involving natural compounds, *in silico* screening, archaeal genetics, and methanogen-targeted vaccines. Their review highlights the need for specificity, safety, cost-effectiveness, and regulatory acceptance.

Malyugina et al. provided a broader review of methane mitigation strategies in ruminant livestock. They synthesized evidence for nutritional additives, seaweeds, tannins, essential oils, probiotics, vaccines, genetic selection, and manure management. A key message from this review is that no single strategy is universally effective. Diet, dose, delivery system, animal species, production stage, and farm context all determine the outcome.

## Beyond the rumen: manure methane and climate vulnerability

Environmental assessment of ruminant systems should not stop at enteric methane. Manure emissions, household vulnerability, and adaptive capacity also shape the real climate impact of livestock production. Two papers in this Research Topic extend the discussion beyond rumen fermentation and show why mitigation must be linked with manure management and farmer resilience. Liu et al. showed that ruminant feces also deserve closer study. Using metagenomic and carbon isotope approaches, they found that sheep feces had a methane production potential more than three times higher than that of non-ruminant feces. Acetoclastic methanogenesis, supported by syntrophic interactions between fermentative bacteria and methanogenic archaea, appeared to be a key driver. These findings expand the mitigation discussion from rumen fermentation to manure management.

Aiswarya et al. shifted the focus from microbial mechanisms to climate vulnerability among buffalo-rearing households in northern and western India. Their composite vulnerability index revealed marked regional differences, with Bihar and Rajasthan showing high vulnerability due to limited adaptive capacity, high sensitivity, and weaker access to veterinary and market services. This paper widens the discussion by showing that climate-smart livestock production must include both emission reduction and farmer resilience.

## Concluding remarks

The 13 articles in this Research Topic highlight that methane mitigation in ruminant systems is not a single-solution problem. Progress will require coordinated attention to rumen microbiology, feed formulation, host factors, manure emissions, and socio-economic resilience. Several approaches show promise, including tannins, seaweeds, monolaurin-based additives, sorghum processing, brewery waste, mushroom residue, rose pomace, and forage–breed management strategies. However, the most useful strategies will be those that reduce methane without compromising feed intake, digestibility, animal performance, product quality, or farmer profitability.

Future research should move beyond descriptive microbiome profiling and focus on functional mechanisms, long-term animal responses, dose optimization, economic feasibility, and on-farm validation. Methane mitigation should not be viewed as a single intervention, but as a system-level goal that links microbial ecology, nutrition, genetics, manure management, and climate adaptation. These papers reinforce the role of ruminant nutrition science in developing lower-emission and more resilient livestock systems.
